# A Female Newborn Infant with FATCO Syndrome Variant (Fibular Hypoplasia, Tibial Campomelia, Oligosyndactyly) – A Case Report

**DOI:** 10.5334/jbr-btr.929

**Published:** 2016-02-26

**Authors:** Gitte Smets, Yoeri Vankan, Annick Demeyere

**Affiliations:** 1 Department of Radiology, Imelda Hospital, Imeldalaan 9, 2820 Bonheiden, Belgium

**Keywords:** Fibular hemimelia, Tibial campomelia, Oligosyndactyly

## Abstract

Congenital limb deficiencies are common birth defects occurring in 1 in 2000 neonates, characterized by the aplasia or hypoplasia of bones of the limbs. Fibular hemimelia is a rare congenital deficiency or absence of the fibula. The disease spectrum ranges from mild fibular hypoplasia to fibular aplasia. Fibular aplasia, tibial campomelia, and oligosyndactyly (FATCO syndrome) are purely descriptive terms for a syndrome of unknown genetic basis and inheritance.

We report on a newborn female with malformations consisting of fibular hypoplasia, tibial campomelia, and oligosyndactyly, a second FATCO variant case. We also review previously reported cases. Given the paucity of reports on this rare syndrome and the lack of a standardized treatment approach, it is important that each case of FATCO syndrome is reported.

## Case Report

We report on a case of fibular hypoplasia, tibial campomelia, and oligodactyly in a female neonate of one day old. The patient was born by elective Cesarean section at 39 weeks, 4 days. She was the first pregnancy of a healthy mother. The infant birth weight was 2660 g, the birth length was 48 cm, and the head circumference at birth was 34 cm. There was no history of prenatal complications. There was no relevant family history. The neonate needed to be insufflated shortly after birth with immediate recuperation.

Physical examination revealed shortening and anterolateral bowing of the left lower limb at the distal third of the tibia with associated overlying soft tissue dimpling and oligosyndactyly of both feet. Only three toes (the fourth and fifth ray were absent) were found on the left extremity and four toes (the fifth ray was absent) on the right extremity.

There was no associated abnormality in the upper limbs. There was no associated facial dysmorphism nor other associated anomalies. Hip ultrasound revealed no congenital hip dysplasia.

**Figure 1:**
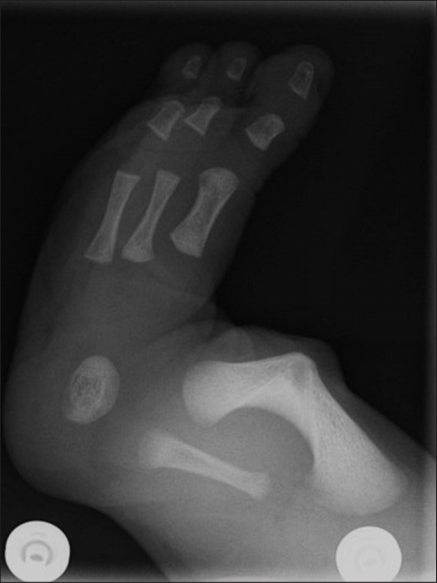


**Figure 2:**
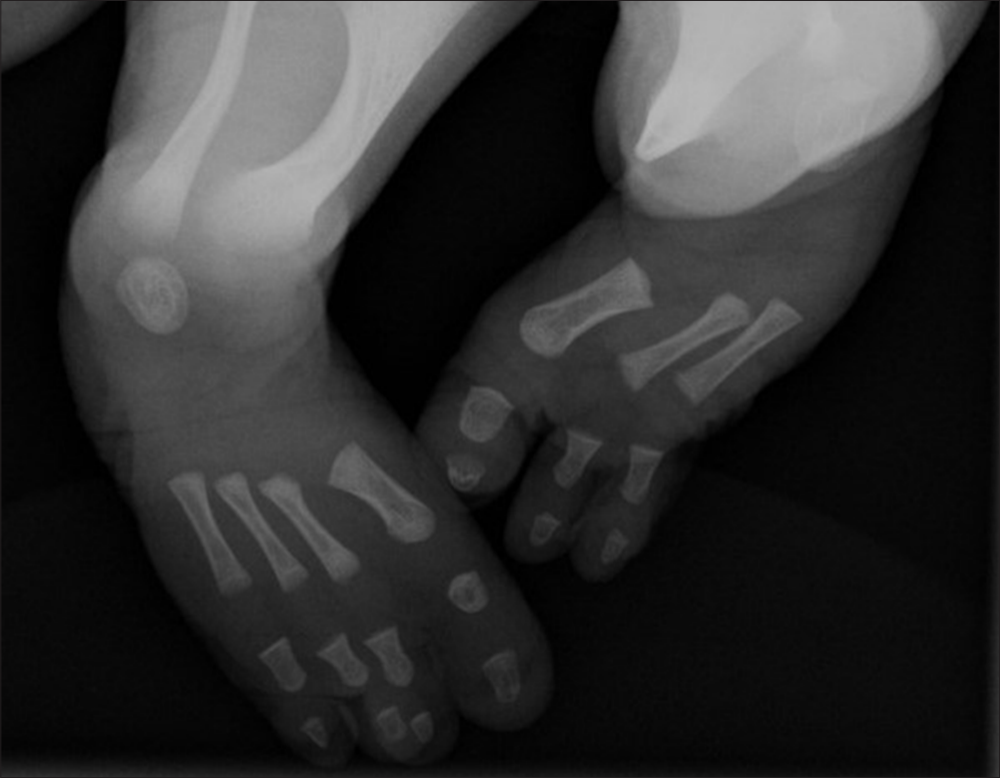


**Figure 3:**
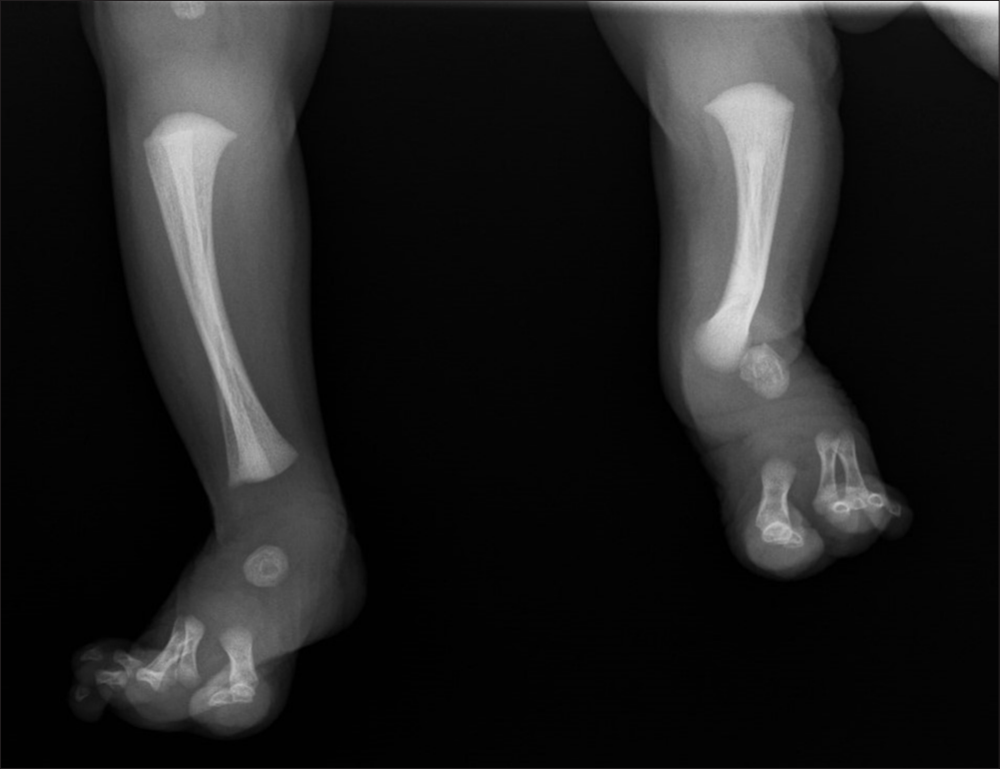


Radiographic examination revealed hypoplasia of the left fibula (Figure 1), absence of two rays left and one ray on the right foot (Figure 2), and anterolateral bowing and shortening of the left tibia (Figure 3). Both femora were normal.

## Discussion

Congenital limb deficiencies are common birth defects, occurring in 1 in 2000 neonates and characterized by the aplasia or hypoplasia of bones of the limbs [1]. Fibular hemimelia (FH) is a rare congenital anomaly and was first described by Gollier in 1698 [2,3]. The term *fibular hemimelia* encompasses a spectrum of disease from mild fibular hypoplasia to fibular aplasia. It has been estimated that there are approximately 5.7 to 20 cases per one million births [4]. FH commonly occurs unilaterally, isolated, and sporadic with unknown cause [2,3]. However, FH may be part of a malformation syndrome. Possible components include femur and tibia shortening, clubfoot, valgus deformity, flexion contracture, and anteroposterior instability of the knee and ankle as well as tarsal coalition with the deficiency of lateral rays of the foot [5]. Even though fibular hemimelia is a rare condition among the long bone deficiency disorders, it is the most common malformation [6].

The precise etiology of FH is unclear. The majority is caused by nongenetic causes such as radiation and teratogens [3]. A proposed theory is that of a disruption of the lower limb developmental field during embryogenesis [3]. The developmental field of the lower extremity includes the pubic portion of the pelvis, proximal femur, patella, anterior cruciate ligament, and lateral or axial foot rays. This developmental field encompasses the commonly associated defects seen with FH, namely, defects of the femur and lateral aspect of the foot.

Courtens et al [2] reported on a male infant with oligosyndactyly of the left hand and the right foot, absence of the right fibula, and anterior bowing of the ipsilateral tibia with associated overlying soft tissue dimpling and reviewed four other cases [2,7,8]. Since all five cases had three major findings in common – fibular agenesis, tibial campomelia, and oligosyndactyly (without heart defect) – they proposed to name it fibular aplasia–tibial campomelia–oligosyndactyly (FATCO) syndrome, which is a purely descriptive term. Thereafter, 8 more patients were documented [9,10,11,12,13,14]. In addition to these 13 patients in whom fibular aplasia was present, Goyal et al [15] introduced a case with unilateral fibular hypoplasia, tibial campomelia, and oligodactyly. Since the classical described case of FATCO involves aplasia of the fibula, Goyal et al [15] named this case *FATCO syndrome variant*. Hence, in 13 of all 14 previously described cases with FATCO syndrome, fibular aplasia was present. Five of these 13 cases presented with bilateral aplasia and 8 with unilateral aplasia in which 3 had fibular aplasia on one side and fibular hypoplasia on the contralateral side [9(case 2)^7^,^12^]. Our case matches the radiological description of a FATCO syndrome variant and is the second report on this variant to the best of our knowledge.

All previously reported cases of FATCO syndrome demonstrated a great clinical variability. The involvement of the upper limb was reported in 9 of 14 cases, of whom 5 cases presented with bilateral and 4 cases with unilateral involvement. Lower limb involvement was reported in all described cases, of whom 8 cases presented with bilateral involvement. In contrast to our case, there was a male sex predilection in all previous reported patients.

Children with FATCO syndrome have a normal mental development and no facial dysmorphism or other anomalies, which is important in counseling for the patient’s family [2,8,10,11,12,14,16]. Two patients thus far have been diagnosed prenatally [13,16] in which nuchal translucency was the earliest manifestation in one case, showing that ultrasonographic examination can lead to an early prenatal diagnosis [13].

The mode of inheritance is still unclear. The syndrome is usually sporadic, but autosomal dominant inheritance with reduced penetration or X-linked inheritance have been suggested by Biegansky et al [9]. Hecht and Scott [7] proposed autosomal recessive inheritance or gonodal mosaicism. However, a genetic basis for this condition remains to be identified. Chromosomal microarray analysis did not show genetic abnormalities in the current case. Furhmann syndrome and Al-Awadi syndrome bear close resemblance to the FATCO syndrome. They are different in terms of the presence of polydactyly, nail dysplasia, and mutations in the *WNT7a*, a gene controlling the dorsovertebral limb development [10]. The previously reported cases were negative for the *WNT7a* mutations [12]. Ectrodactyly and fibular a/hypoplasia share the full phenotypic spectrum of FATCO syndrome; whether they are allelic disorders or represent two variable presentations in the spectrum of the same disorder is not an established fact.

The preservation of the foot and equalization of the length of extremities is the most desirable goal of treatment. The therapies for FH are surgical and include limb-lengthening procedures, epiphysiodesis, and amputation with prosthesis. The decision to proceed with one or the other is usually individualized from case to case. In cases in which there is a nonfunctional foot and more than 50 per cent shortening at birth in comparison with the expected length, surgical Syme’s or Boyd’s amputation with early use of a prosthesis should be performed [17]. The combination of epiphysiodesis with elongation produces the best outcome and is best accepted by the patients [17]. In our case, multidisciplinary consult has led to the conclusion to wait until the patient has reached the age of one year and then reevaluate the options.

## Competing Interest

The authors declare that they have no competing interests.
